# A Novel Feedforward Model of Piezoelectric Actuator for Precision Rapid Cutting

**DOI:** 10.3390/ma16062271

**Published:** 2023-03-11

**Authors:** Bowen Zhong, Shilin Liu, Chenjun Wang, Ziqi Jin, Lining Sun

**Affiliations:** College of Mechanical and Electrical Engineering, Soochow University, Suzhou 215137, China

**Keywords:** piezoelectric actuators, precision cutting, asymmetrical hysteresis, cutting accuracy, self-adaptive cooperative PSO algorithm

## Abstract

The piezoelectric actuator has been widely used in modern precision cutting technology due to its fast response speed and high positioning accuracy. In recent years, with the development of precision technology, modern cutting requires higher and higher cutting accuracy and efficiency. Therefore, this paper proposes a feedforward control method based on the modified Bouc–Wen (MBW) model. Firstly, a novel asymmetrical modified Bouc–Wen model with an innovative form of shape control function is developed to model the hysteresis nonlinearity property of piezoelectric actuators. Then, a self-adaptive cooperative particle swarm optimization (PSO) algorithm is developed to identify the parameters of MBW model. The comparative evaluation reveals that the MBW model outperforms the classical Bouc–Wen (CBW) model by 66.4% in modeling accuracy. Compared with traditional PSO algorithm, the self-adaptive cooperative PSO algorithm can obtain minimum fitness in parameter identification. Furthermore, the feedforward control strategy is realized to improve the position tracking accuracy. A position tracking experiment verifies that the feedforward control strategy improves the tracking accuracy of piezoelectric actuators significantly compared with the open-loop control strategy.

## 1. Introduction

Piezoelectric actuators (PZAs) have been widely used in various ultra-precision cutting technologies, such as ultrasonic vibration cutting [1,2], fast tool servo technology (FTS) [3], ultra-precision cutting technology [4] and even machining vibration detection [5], due to their high positioning resolution, small size and quick response [6,7,8]. Especially with the rapid development of three-dimensional elliptical vibration cutting (3D-EVC) [9,10] in the past few years, 3D-EVC not only has the advantages of restraining tool wear, restraining tool brittleness and obtaining excellent machining quality, but also has obtained higher machining efficiency. However, correspondingly, the requirements for cutting accuracy are also getting higher and higher.

In order to achieve good positioning performance of piezoelectric ceramics and ensure the positioning accuracy in the cutting process, Shamoto, et al. [11] have realized a new ultrasonic vibration controller for ultrasonic elliptical vibration cutting through feedback sensors. However, the control algorithm of this method is complex and the control time is long, which affects the processing efficiency. While the pure open-loop control method can effectively reduce the control time, the inherent nonlinear hysteresis of PZA will significantly reduce the positioning accuracy [12], which will have a negative impact on the cutting accuracy. In order to ensure cutting efficiency and improve cutting accuracy, feedforward control has become the most common and effective control method [13]. The main idea of feedforward control is to develop a mathematical model to characterize the complex hysteresis of PZAs, then to implement a feedforward compensator based on the inverse hysteresis model, so that the response of PZAs can be linearized [14]. Arindam Bhattacharjee et al. [15] use the Preisach model, which mainly uses multiple Preisach operators and weighted superposition to describe the hysteresis characteristics; Kim et al. [16] used the Bouc–Wen model to describe the relationship between the restoring force and displacement of the hysteresis system to describe the piezoelectric hysteresis characteristics; Naser M. F. et al. [17] used the Duhem model, which has clear equations. The hysteresis nonlinearity can be described by adjusting the parameters of the equation; Qing et al. [18] proposes a PI mathematical model based on the improvement of the traditional Preisach model to describe the hysteresis characteristics of the piezoelectric actuator. The above works have improved the modeling accuracy to a certain extent, and always have lots of parameters need to determine. Zhu and Wang [19] took a unique mathematical method for parameter identification, but the processes is quite complex and inefficient. Some optimization algorithms such as Particle Swarm Optimization (PSO) and Genetic Algorithm (GA) are also used for parameter identification of hysteresis model; however, these methods are easy to fall into local extremum [20].

In this paper, a hysteresis model-based feedforward control method is proposed to improve the position tracking accuracy. Consequently, a modified Bouc–Wen (MBW) model based on shape control function theory [21] is proposed for characterizing the asymmetrical hysteresis behavior of PZAs. The main novelty of this model is modifying the shape control function with the first and second differential of the input, so that the shape control function of MBW model has more degrees of freedom than that of CBW model. A self-adaptive cooperative PSO algorithm is proposed to identify the parameters of MBW models. The performance of MBW model and self-adaptive cooperative PSO algorithm is evaluated. Then, the MBW model-based feedforward controller is designed to improve tracking performance. The tracking performance of proposed control strategy is verified by experiment.

The rest of this paper is organized as follows. Section 2 proposes the MBW model. Section 3 proposes a novel modified PSO algorithm utilized to identify the parameters of MBW model. Section 4 evaluates the performance of the proposed MBW model and modified PSO algorithm. Section 5 finishes the displacement tracking experimental utilizing feedforward control strategy based on the MBW model. At the end, conclusions are provided in Section 6.

## 2. MBW Model for PZAs

### 2.1. Hysteresis Analysis

**Figure 1:**
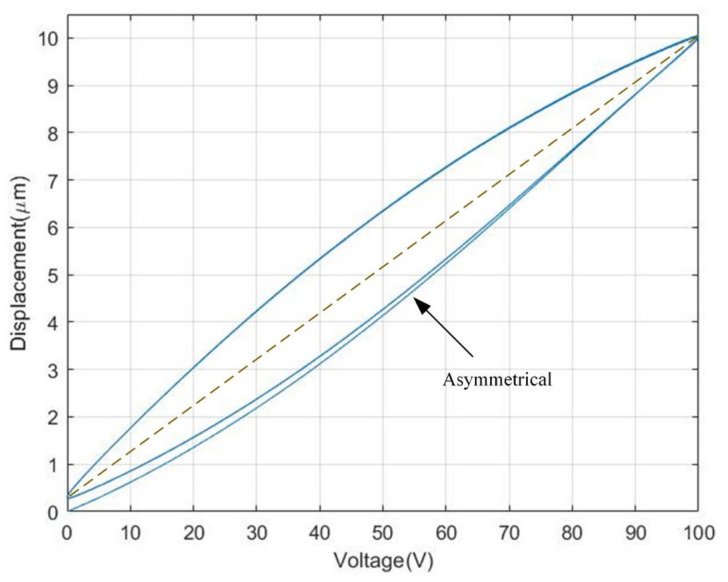
Measured hysteresis loop of a PZA.

Figure 1 is the measured curve of a piezoelectric actuator under the input signal of a 0.1 Hz sinusoidal voltage. As can be seen from Figure 1, the relationship curve between the input voltage and output displacement of PZA is a nonlinear asymmetrical curve. Considering the output displacement of a PZA is constructed of a linear component x(t) and a nonlinear hysteresis component h(t), the relationship between the output displacement y(t) and the input voltage u(t) can be expressed as:

(1) $$\{\begin{array}{c} y(t)=x(t)-h(t)\\ x(t)=ku(t) \end{array},$$

**Figure 2:**
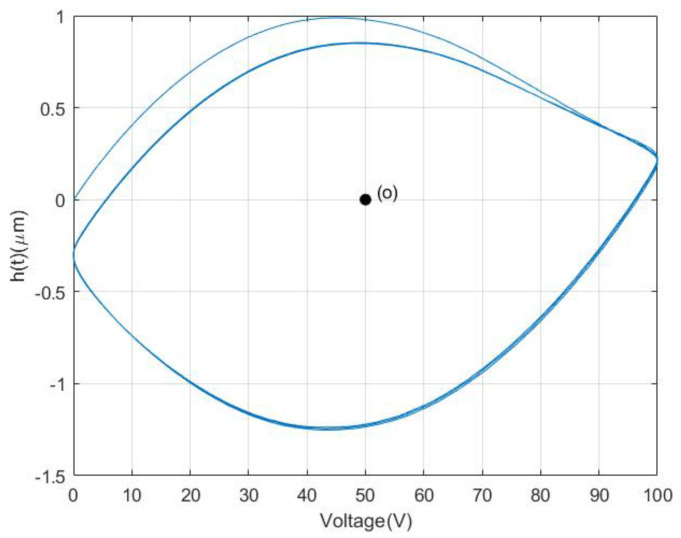
Nonlinear components of PZA.

For the nonlinear part, a least-squares method can be taken to separate the nonlinear component h(t) which is shown in Figure 2.

From Figure 2, it can be seen that the nonlinear component is asymmetrical about the central point o, which result in the asymmetrical hysteresis property of PZAs.

### 2.2. CBW Model

**Figure 3:**
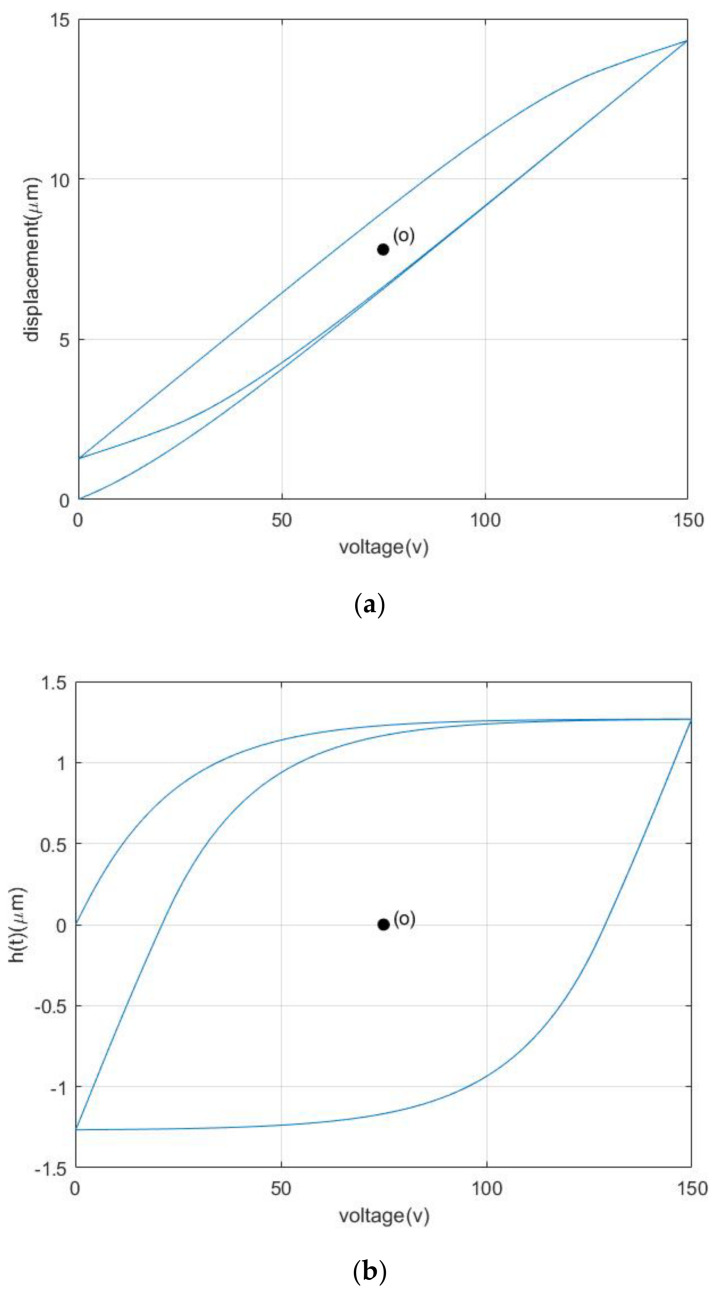
Output displacement and nonlinear components of Bouc−Wen model. (**a**) Output displacement of Bouc−Wen model. (**b**) Nonlinear component of Bouc−Wen model.

The CBW model takes a nonlinear differential equation [19] to describe the nonlinear hysteresis property. It can be mathematically expressed as follows:

(2) $$\{\begin{array}{l} y(t)=x(t)-h(t)\\ x(t)=ku(t)\\ \dot{h}(t)=A\dot{u}(t)-\beta |\dot{u}(t)||h(t){|}^{n-1}h(t)-\gamma \dot{u}(t)|h(t){|}^{n}, \end{array}$$

where x(t) and h(t) are both the function of the input voltage u(t), y(t) denotes the output displacement of the PZA, and $\dot{h}(t)$ and $\dot{u}(t)$ are, respectively, the derivative of the nonlinear hysteresis component h(t) and input voltage u(t) with respect to the time. k,A,∂,β,γ,n are the parameters of the CBW model, and n=1 is often chosen for characterizing hysteresis property of PZAs [22]. The simulation of the CBW model with a group of given parameters (k=0.104,A=0.057,γ=0.018,β=0.027,n=1) is shown in Figure 3.

From Figure 3, it can be obviously seen that both the nonlinear component and output displacement generated by the CBW model is symmetrical about the central point o.

The nonlinear differential equation in Equation (2) can be equivalently rewritten as follows:

(3) $$\dot{h}(t)=\dot{u}(t)\{A-[\beta \mathrm{sgn}(h(t)•\dot{u}(t))+\gamma ]|h(t){|}^{n}\},$$

where sgn is the signum function: $\mathrm{sgn}(x)=\{\begin{array}{lll} 1 & & x>0\\ -1 & & x<0 \end{array}$. Equation (3) can be further rewritten as follows:

(4) $$\{\begin{array}{l} \dot{h}(t)=\dot{u}(t)\{A-\psi (\dot{u},h)|h(t){|}^{n}\}\\ \psi (\dot{u},h)=\gamma +\beta \mathrm{sgn}(\dot{u}(t)h(t)), \end{array}$$

**Figure 4:**
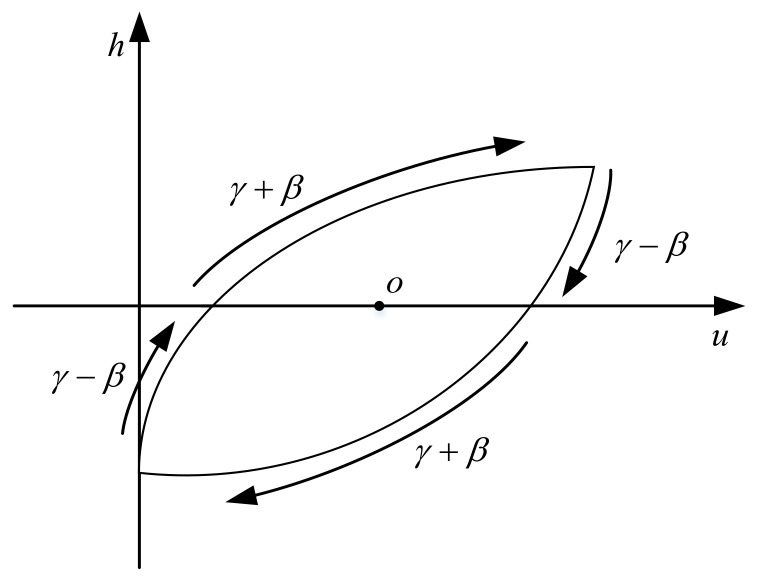
Values of shape control function for Bouc–Wen model.

$\psi (\dot{u},h)$ in Equation (4) is the shape control function of CBW model and the value of $\psi (\dot{u},h)$ controls the shape of the hysteresis loop of CBW model. The function $\psi (\dot{u},h)$ can have four different phases determined by the signs of $\dot{u},h$. As is shown in Figure 4, $\psi (\dot{u},h)$ can have only two independent values in the four phases, which is symmetrical about the central point o. Therefore, the CBW model contains only two degrees of freedom for controlling the shape of the hysteresis loop; this is the source reason of its symmetrical property.

### 2.3. MBW Model

To model the hysteresis loop of PZAs with an asymmetrical model, the shape control function of Bouc–Wen model must be modified to take independent values in all phases. Considering that the hysteresis loop shape of PZAs is related to the rate of input signal; the second-order differential of the input voltage $\ddot{u}$ is introduced to the shape control function of MBW model as follows in this paper.

(5) $$\begin{array}{ll} \psi (\dot{u},\ddot{u},h)= & \gamma +\beta_{1}\mathrm{sgn}(\dot{u}h)+\beta_{2}\mathrm{sgn}(\dot{u}\ddot{u})+\beta_{3}\mathrm{sgn}(\ddot{u}h)\\ & +\beta_{4}\mathrm{sgn}(\dot{u})+\beta_{5}\mathrm{sgn}(h)+\beta_{6}\mathrm{sgn}(\ddot{u})\;\;\;, \end{array}$$

where $\gamma ,\beta_{1},\ldots ,\beta_{6}$ are fixed parameters of the model.

**Figure 5:**
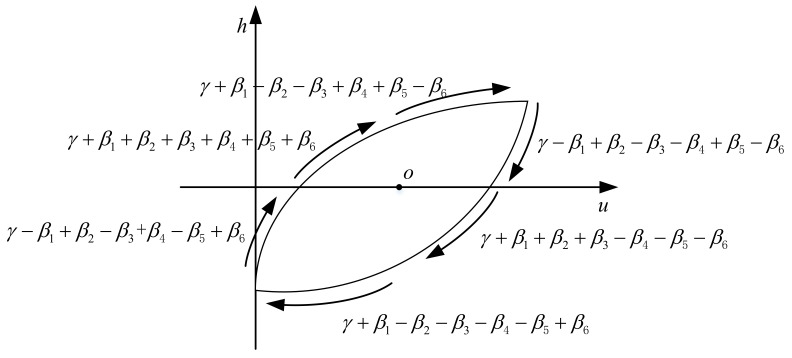
Values of modified shape control function.

Under the sinusoidal voltage input signal, the shape control function in Equation (5) has six phases and independent values determined by the combinations of signs of $\dot{u},\ddot{u},h$ in each phase as shown as Figure 5. The value of modified shape control function $\psi (\dot{u},\ddot{u},h)$ and its correspondence to the signs combinations of $\dot{u},\ddot{u},h$ for six different phases is listed in the Table 1.

By use of above-mentioned shape control function, the modified model can be written as follows.

(6) $$\left\{ \begin{array}{l} x(t)=ku(t)-h(t)\\ \dot{h}=\dot{u}(t)\{A-|h(t)|\psi (\dot{u},\ddot{u},h)\}\\ \psi (\dot{u},\ddot{u},h)=\gamma +\beta_{1}\mathrm{sgn}(\dot{u}h)+\beta_{2}\mathrm{sgn}(\dot{u}\ddot{u})+\beta_{3}\mathrm{sgn}(\ddot{u}h)+\beta_{4}\mathrm{sgn}(\dot{u})+\beta_{5}\mathrm{sgn}(h)+\beta_{6}\mathrm{sgn}(\ddot{u}), \end{array}\right.$$

**Figure 6:**
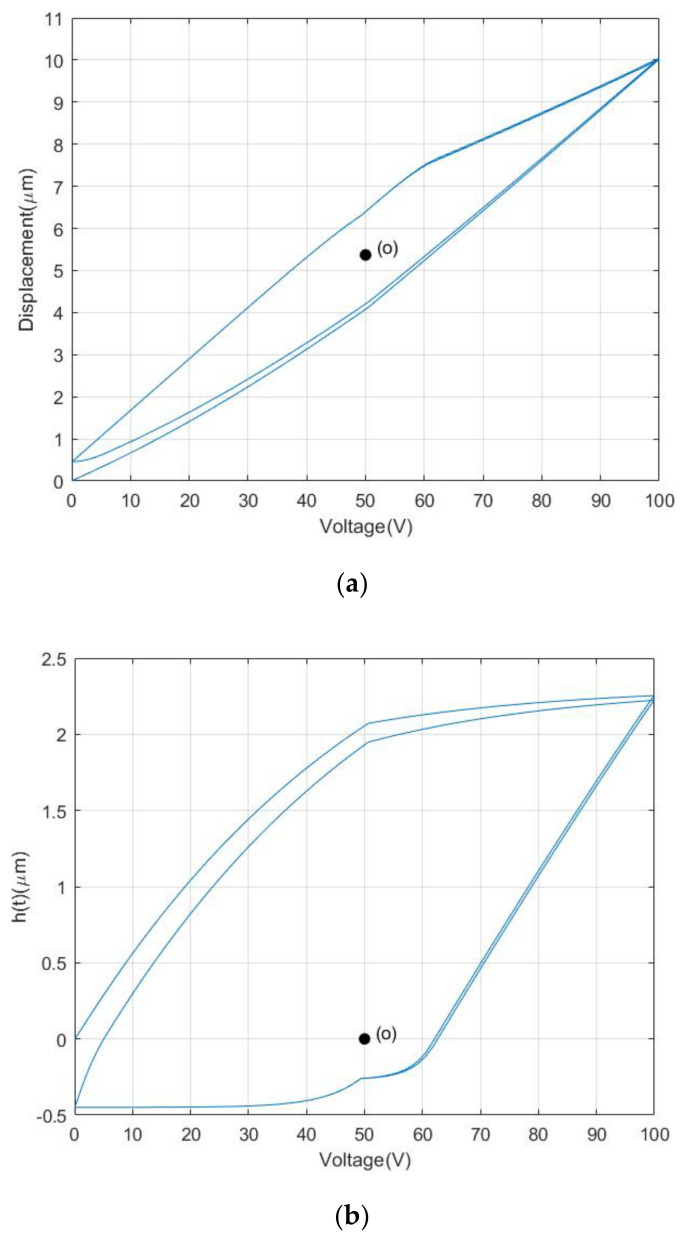
Output displacement and nonlinear components of MBW model. (**a**) Output displacement of MBW model. (**b**) Nonlinear component of MBW model.

Figure 6 shows the simulation of the MBW model with a group of given parameters (k=0.123,A=0.063,γ=0.057, $\beta_{1}=0.049,\beta_{2}=-0.028,\beta_{3}=0.050$, $\beta_{4}=-0.065$, $\beta_{5}=-0.026,\beta_{6}=-0.018,n=1$).

It is shown obviously in Figure 6 that both the nonlinear component and output displacement generated by the MBW model is asymmetrical about the central point o. Therefore, the MBW model proposed in this paper indeed plays the role of asymmetrical property and can be used for modeling the asymmetrical hysteresis property of PZAs.

## 3. Parameters Identification Using a Novel Modified PSO Algorithms

### 3.1. Parameters Identification with Traditional PSO Algorithm

PSO algorithm is an optimization algorithm inspired by the flock of birds’ preying behavior. PSO algorithm starts with a swarm of particles that are randomly distributed in D-dimensional search space. Each particle can be described by two D-dimensional vectors: the position vector $P_{i}^{t}=\{p_{i1}^{t},p_{i2}^{t}\cdots p_{iD}^{t}\}$ and the velocity vector $V_{i}^{t}=\{v_{i1}^{t},v_{i2}^{t}\cdots v_{iD}^{t}\}$, where i is the particle number and t is the iteration generation number. During the iteration, each particle updates its position and velocity by tracking the individual past best position $Pbest_{i}=(pbest_{i1},pbest_{i2}\ldots pbest_{iD})$ and global past best position $gbest=(gbest_{1},gbest_{2}\ldots gbest_{D})$. The updating rule of the position vector and the velocity vector can be written as the following two equations [23]:

(7) $$\begin{array}{l} V_{i}^{t+1}=\omega V_{i}^{t}+c_{1}\times r_{1}\times (Pbest_{i}-P_{i}^{t})+c_{2}\times r_{2}\times (gbest-P_{i}^{t})\\ P_{i}^{t+1}=P_{i}^{t}+V_{i}^{t+1}, \end{array}$$

where ω is the inertia weight, and $c_{1},c_{2}$ are the acceleration constants, $r_{1},r_{2}$ are the random numbers in range of 0 to 1. In general, $c_{1}=c_{2}=1.49445$ is chosen for the updating and ω is set to a fixed value or decrease linearly as shown as Equation (8) during the iteration, which is called linear “single variable with iteration” inertia weight [24].

(8) $$\omega =\omega_{\max}-\frac{\left(\omega_{\max}-\omega_{\min}\right)}{iter_{\max}}\times iter,$$

where $iter_{\max}$ is the maximum of iterations generations, and iter is the current generation.

The fitness of each particle is evaluated by the fitness function and the root mean squared error is chosen as the fitness function. It is shown as follows:

(9) $$f(k,A,\gamma ,\beta_{1},\beta_{2},\beta_{3},\beta_{4},\beta_{5},\beta_{6})=J=\sqrt{\frac{1}{N}\sum_{i=1}^{N}{\left(y(i)-\hat{y}(i)\right)}^{2}},$$

In Equation (9), N is the sample size, and y(i) and $\hat{y}(i)$ are, respectively, the output displacement of the PZA and the predict displacement of the MBW model at ith sampling point.

Thus, the parameter identification procedure with traditional PSO algorithm is illustrated as follows:

*Step 1:* Initialize the PSO population with randomly position and velocity in the searching space.

*Step 2:* For each particle i, evaluate the fitness using Equation (9).

*Step 3:* Replace $Pbest_{i}$ with the particle i if the particle’s fitness is smaller than its $Pbest_{i}$. Replace gbest with the particle i if the particle’s fitness is smaller than its gbest.

*Step 4:* Update the velocity and position of each particle based on Equations (7) and (8).

*Step 5:* Repeat steps 2–4 until the maximum iteration generations or minimum error is reached.

### 3.2. Parameters Identification with Self-Adaptive Cooperative PSO Algorithm

According to the velocity updating rule shown in Equation (7), a new particle inherits its velocity from the last generation and learns from the individual past best position and global past best position. From Equation (7), it is clear that the inertia weight ω controls the impact of last generation velocity on the current generation one. A larger inertia weight ω helps the PSO population with global exploration, a smaller inertia weight ω helps with local searching [25].

The linear decreasing inertia weight strategy in traditional PSO algorithm simply adopts a larger inertia weight at early phase of iteration and a smaller one at later phase of iteration, which leads traditional PSO to easily fall into local optimum. In this paper, a kind of self-adaptive inertia weight strategy is proposed, and can be written as follows.

(10) $$\omega =\{\begin{array}{l} \omega_{\min}+\frac{\left(\omega_{\max}-\omega_{\min})(f_{i}-f_{\min}\right)}{f_{avg}-f_{\min}},f\leq f_{avg}\\ \omega_{\max},\;\;\;\;\;\;\;f>f_{avg}, \end{array}$$

where $f_{\min},f_{avg},f_{i}$ are, respectively, the minimum fitness, average fitness and particle fitness. When the particle fitness is better than average fitness, ω will adopt a small value to keep local search ability. When the particle is worse than average fitness, ω will adopt the maximum inertia weight to have a large updating step so that the particle will tend to explore larger space.

As can be seen from Equation (6), there are nine parameters $(k,A,\gamma ,\beta_{1},\beta_{2},\beta_{3},\beta_{4}$ $,\beta_{5},\beta_{6})$ in the MBW model proposed in this paper. In traditional PSO algorithm, one swarm that is composed of a population of nine-dimensional vectors is used for optimization, the position of every particle is a nine-dimensional vector that can be used as a potential solution. Every time updating the particle, nine dimensions of the position vector will update together. This allows for a large possibility that some dimensions of the vector moved closer to the best solution while some dimensions moved further from the best solution. To overcome this undesirable behavior of traditional PSO algorithm, a cooperative approach for PSO algorithm is proposed in this paper. The cooperative approach for PSO algorithm divides a potential solution (nine-dimensional vector) of the MBW model into three three-dimensional vector. It utilizes three swarms for optimization, and each swarm is composed of a population with three-dimensional vectors. The particles of every swarm update their position individually and evaluate its fitness by combination with the same serial number particle of other two swarms during the optimizing duration. This kind of approach can greatly decrease the possibility of some dimensions of the particle going forward while others going backward.

From the description above, the parameter identification processes with self-adaptive cooperative PSO algorithm can be illustrated as follows:

*Step 1:* Initialize three PSO subpopulations with randomly position and velocity in the three-dimensional sub searching space of each swarm.

*Step 2:* Construct the particles of each swarm in sequence into a nine-dimensional vector, which is a potential solution of the MBW model. 

*Step 3:* Evaluate the fitness of the combined nine-dimensional vector (potential solution) using Equation (9). The fitness of each potential solution is regarded as the fitness of the particle with same sequence number in three sub particle swarms.

*Step 4:* For each swarm, replace $Pbest_{i}^{k}(k=1,2,3)$ with the particle i if the particle’s fitness is smaller than its $Pbest_{i}^{k}(k=1,2,3)$. Replace $gbest^{k}$ with the particle i if the particle’s fitness is smaller than its $gbest^{k}$.

*Step 5:* For each swarm, update the velocity and position of each particle based on Equations (7) and (8).

*Step 6:* Repeat steps 2–5 until the maximum iteration generations or minimum error is reached.

## 4. Model Parameters Identification and Algorithm Evaluation

### 4.1. Hysteresis Model Evaluation

In order to evaluate the hysteresis modeling performance of different model, the CBW model and MBW model is taken to expect the response of the piezoelectric actuator under 0.1 Hz sinusoidal voltage, as shown in Figure 1a and the parameters of each model is identified utilizing the self-adaptive cooperative PSO algorithm mentioned above. The root mean square error (RMSE) and maximum error ($err_{\max}$) are taken to evaluate the modeling accuracy of different model.

(11) $$RMSE=\sqrt{\frac{1}{N}\sum_{i=1}^{N}{\left(y(i)-\hat{y}(i)\right)}^{2},}$$

(12) $$err_{\max}=\max(y(i)-\hat{y}(i)),$$

**Figure 7:**
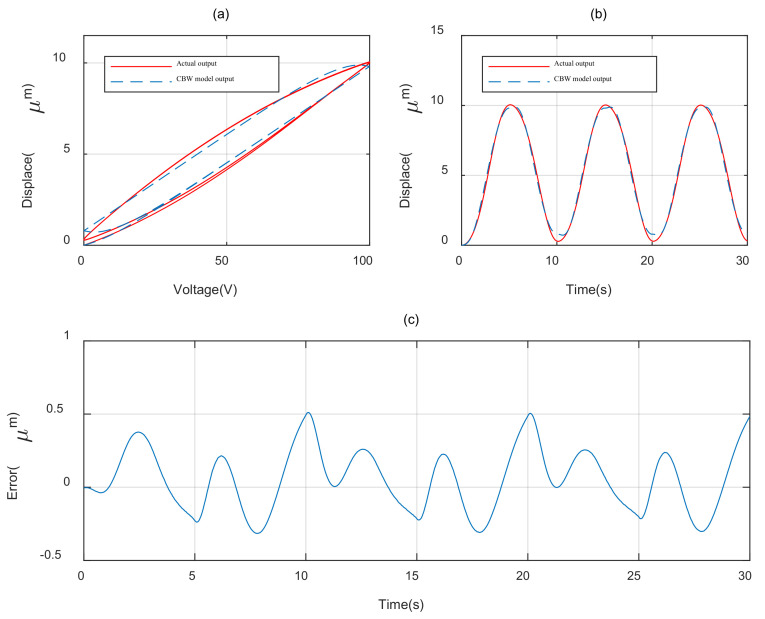
Modeling result of CBW model. (**a**) Hysteresis loop. (**b**) Displacement curve. (**c**) Error curve.

**Figure 8:**
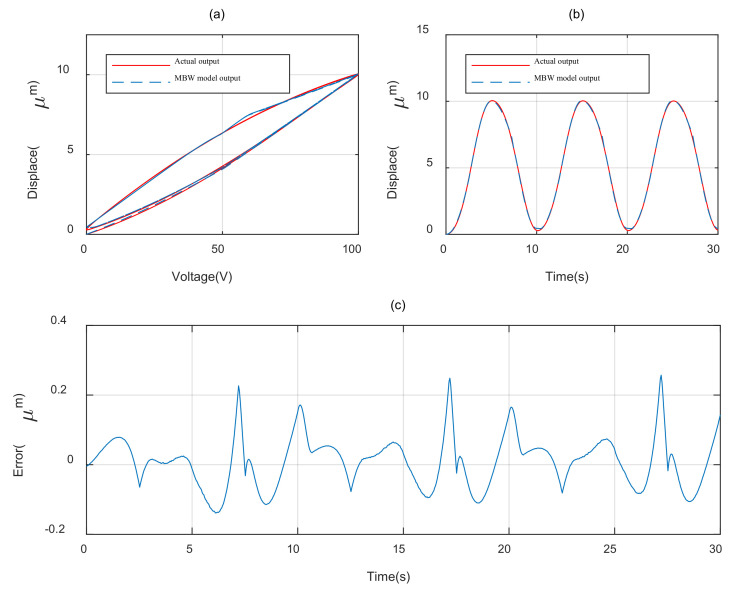
Modeling result of MBW model. (**a**) Hysteresis loop. (**b**) Displacement curve. (**c**) Error curve.

**Figure 9:**
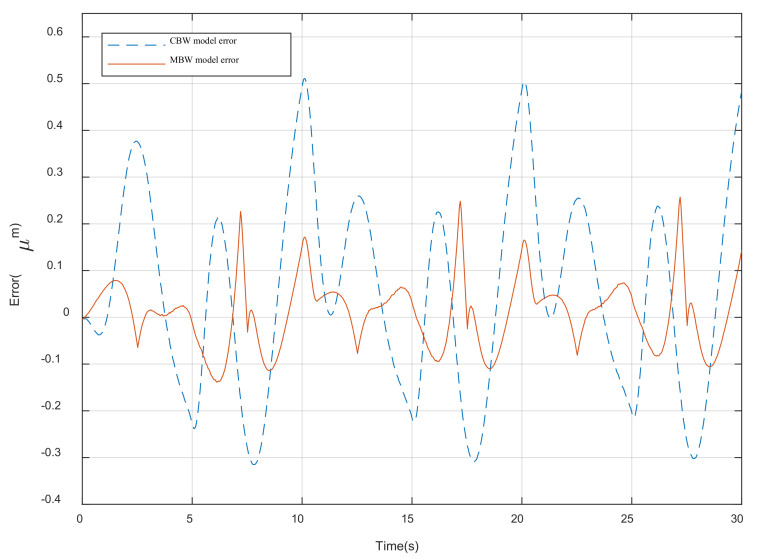
Modeling error of MBW model.

The parameter identification results of the different model are, respectively, shown in Table 2 and Table 3. The modeling results with CBW model and MBW model are illustrated, respectively, in Figure 7 and Figure 8. The modeling error comparisons of the two different model are shown in Figure 9. Furthermore, the quantitative evaluation of each model utilizing Equations (11) and (12) is shown in Table 4.

It can be concluded from Figure 7, Figure 8 and Figure 9 and Table 3 that the MBW model proposed in this paper perform more accurately than CBW model in modeling the hysteresis characterize of PZAs. Compared with the CBW model, the RMSE of MBW model is reduced 66.4% and the maximum error is reduced 49.7%.

### 4.2. Parameter Identification Algorithm Evaluation

**Figure 10:**
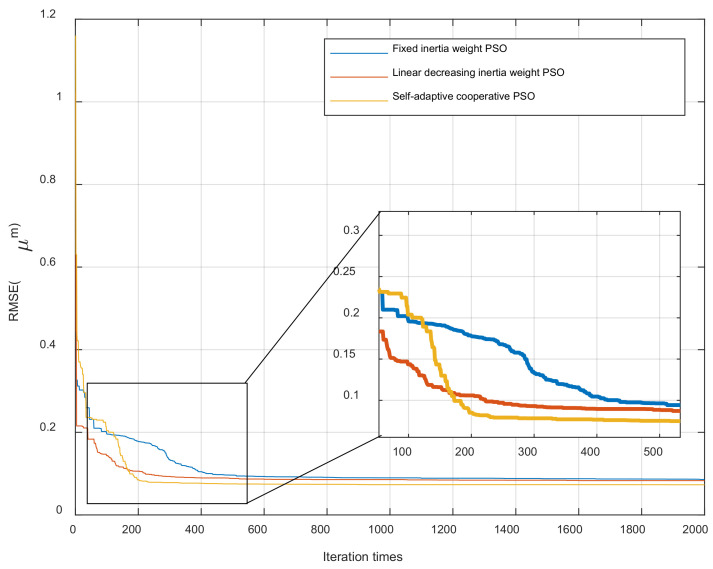
Convergence process of different algorithms.

In order to evaluate the performance of proposed modified PSO algorithm (self-adaptive cooperative PSO) in parameter identification. The parameter identification experiment for MBW model utilizing traditional PSO algorithm (fixed inertia weight and linear decreased inertia weight) and modified PSO algorithm is executed, respectively. For all experiments, the same fitness function as Equation (9) and the same searching space are used. The parameters identification result with different algorithms are listed in Table 5 and the fitness function convergence process of each algorithm is shown in in Figure 10.

It can be seen from Table 5 and Figure 10 that the modified PSO algorithm outperforms other algorithms with best accuracy.

**Figure 11:**
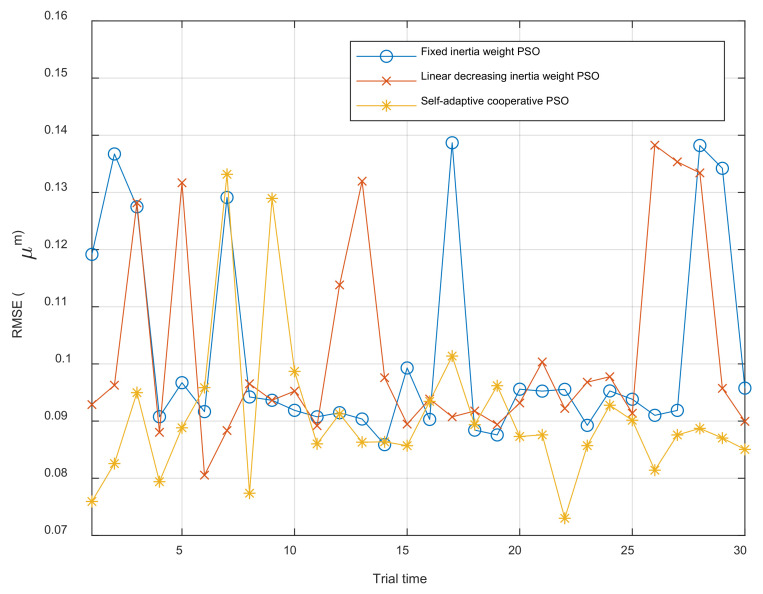
Comparison of 30-times repeat experiments with different algorithm.

Considering that there are random factors inside the optimization algorithms, the performance of the algorithm cannot be judged by a single trial. Therefore, the parameter identification experiments with different algorithms are repeated 30 times, respectively. The maximum, minimum and average fitness J of each algorithm is listed in Table 6. And 30 fitness results for each algorithm are illustrated in Figure 11.

The result of repeat experiment demonstrates that the modified PSO algorithm proposed in this paper can obtain the minimum fitness J compared with traditional PSO algorithm, which means the MBW model can obtain best parameters with self-adaptive cooperative PSO algorithm used for parameter identification.

## 5. Feedforward Control Experiment

### 5.1. Experiment Setup

**Figure 12:**
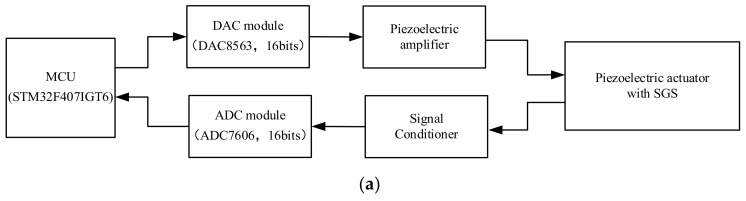

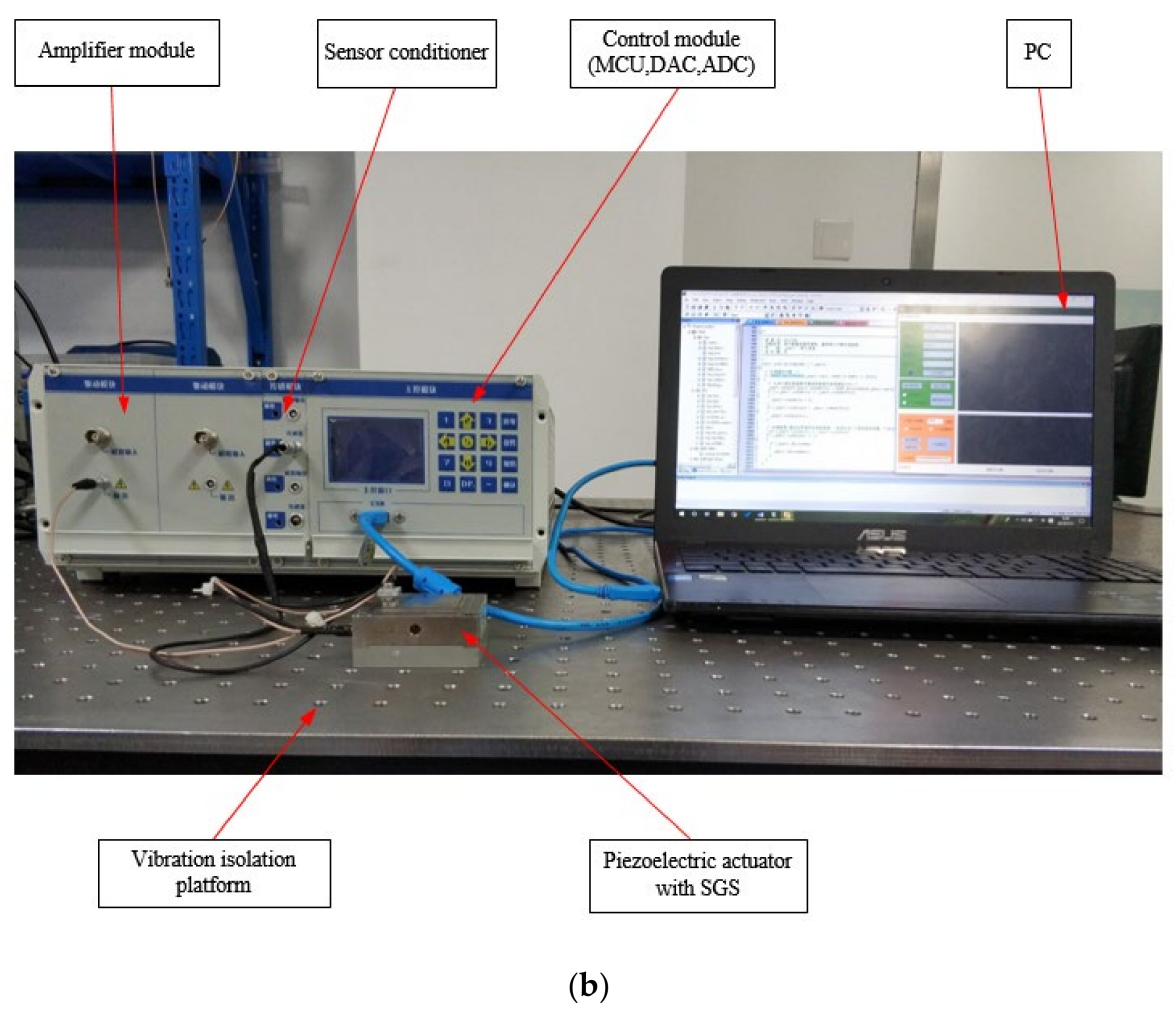
Experiment system for PZA. (**a**) Schematic of experiment system. (**b**) Picture of experiment system.

The experiment for feedforward control is executed on a PZA MPT-1JRL002 (Micro, Suzhou, China) nanometer positioning stage. Figure 12a shows the schematic of experiment system and Figure 12b is the picture of the experiment system. As can be seen from Figure 12, the positioning stage is controlled by a precision positioning controller, which is constructed mainly by control module, amplifier module and sensor conditioner. The digital control signal is sent by the MCU(STM32-F407IGT6) (STMicroelectronics, Shanghai, China) in the control module, then the signal is converted to analog signal by the DA converter (DAC8563 16 bits) (DA converter Texas Instruments, Shanghai, China) and amplified 15 times by the amplifier module. The amplified voltage is applied to PZA and its displacement is measured by a resistance strain gauge sensor installed within the PZA as a nanometer displacement sensor. The sensor signal is acquired by an AD converter (ADC7606 16bits) (Analog Devices, Shanghai, China) and then transmitted to the MCU.

### 5.2. Feedforward Compensator Based on MBW Model

According to the MBW model proposed in Section 3, the output displacement of a PZA can be illustrated as follows:

(13) x(t)=ku(t)−h(t),

Therefore, the feedforward hysteresis compensator can be directly written as follows [12]:

(14) $$u(t)=\frac{1}{k}(x_{d}(t)+h(u(t))),$$

where the desired displacement $x_{d}(t)$ is taken to take place of the output displacement x(t) and it is regarded as input in Equation (14). u(t) is the output of the hysteresis compensator and h is a function of u. However, Equation (14) cannot be solved because term h(⋅) involves u(t). To address this problem, u(t−ΔT) is taken to replace u(t) approximately in term h(⋅) where ΔT is the sample interval. Thus, the output u(t) can be solved and Equation (14) can be rewritten as follows:

(15) $$u(t)=\frac{1}{k}(x_{d}(t)+h(u(t-\Delta T)))$$

**Figure 13:**
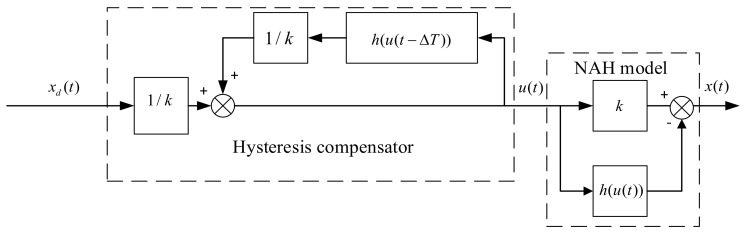
Block diagram of hysteresis compensator based on MBW model.

The block diagram of feedforward compensator expressed in Equation (15) is shown as Figure 13.

### 5.3. Control Experiment

**Figure 14:**
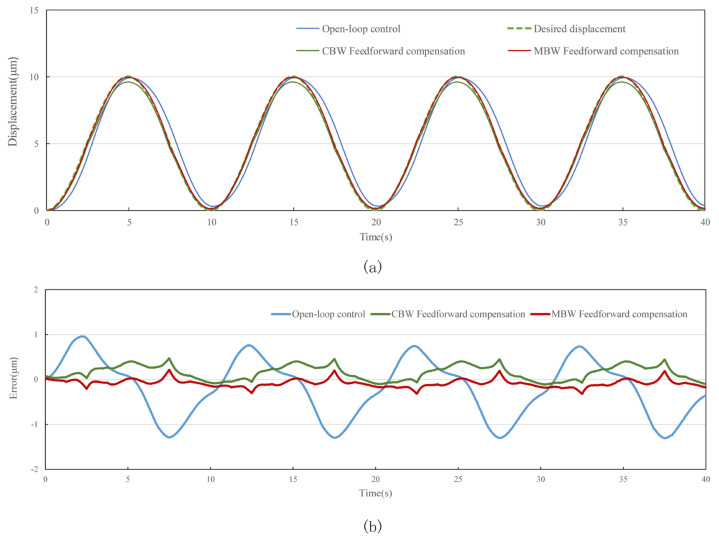
Standard sinusoid signal tracking performance with different control method at 0.1 Hz. (**a**) tracking displacement. (**b**) error curve.

**Figure 15:**
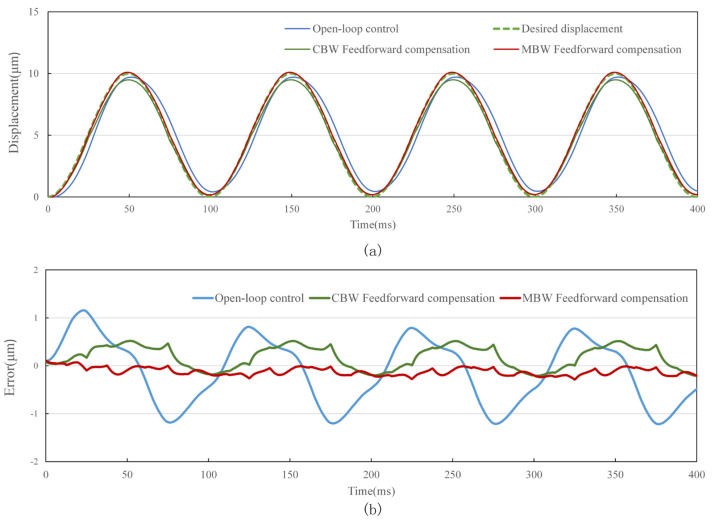
Standard sinusoid signal tracking performance with different control method at 10 Hz. (**a**) tracking displacement. (**b**) error curve.

**Figure 16:**
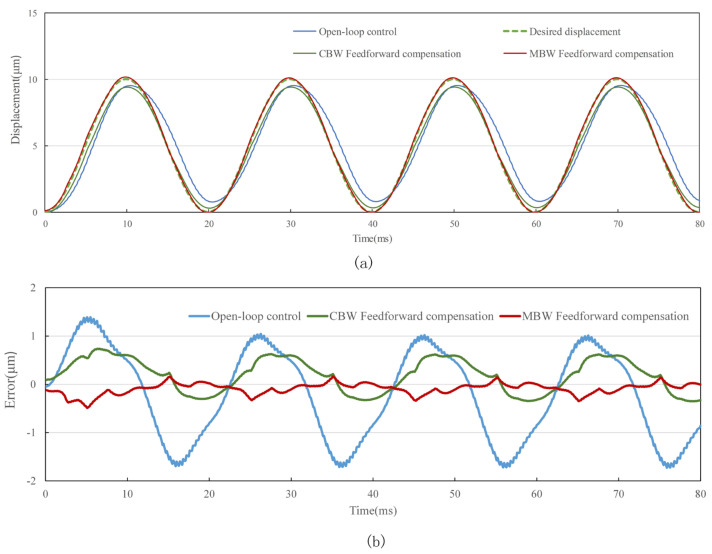
Standard sinusoid signal tracking performance with different control method at 50 Hz. (**a**) tracking displacement. (**b**) error curve.

**Figure 17:**
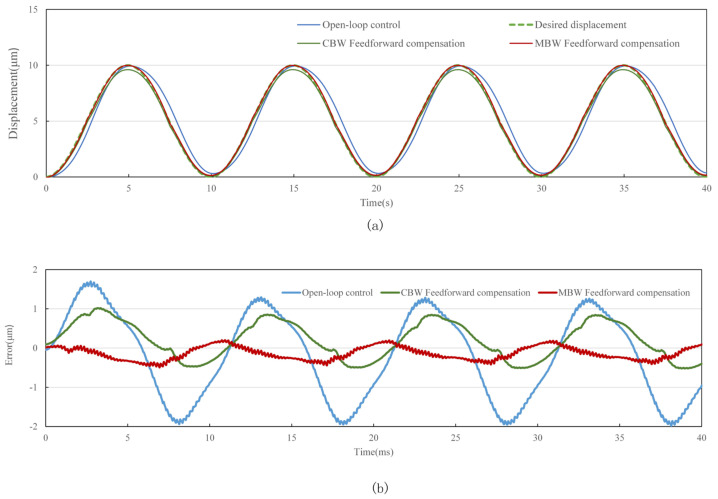
Standard sinusoid signal tracking performance with different control method at 100 Hz. (**a**) tracking displacement. (**b**) error curve.

The standard sine signal with frequency of 0.1 Hz, 10 Hz, 50 Hz and 100 Hz and amplitude of 10 μm is used as the target of the tracking experiment. In order to evaluate the performance of feedforward control strategy, tracking experiments using open-loop control, CBW feedforward control and MBW feedforward control are carried out on the experimental system shown in Figure 14, Figure 15, Figure 16 and Figure 17, and the experimental errors of different control methods are calculated.

As shown in the figure above and Table 7, compared with open-loop control and CBW feedforward control, MBW feedforward control method has significantly improved the tracking performance of sinusoidal waveform, especially at high frequencies of 50 Hz and 100 Hz. The RMSE of MBW feedforward control reaches 0.147 μm and 0.236 μm in tracking standard sinusoid signal at the frequency of 50 Hz and 100 Hz, respectively, while that of the CBW feedforward control yields 0.391 μm and 0.501 μm, respectively. Compared with CBW feedforward control, the feedforward control reduces RMSE by 37.6% and 60.3% in tracking standard sinusoid signal at the frequency of 50 Hz and 100 Hz, respectively.

## 6. Conclusions

In this paper, a novel asymmetrical modified Bouc–Wen (MBW) model is proposed to simulate the hysteresis characteristics of PZA by revise the shape control function of classical Bouc–Wen (CBW) model in order to serve the precision cutting technology. A self-adaptive cooperative PSO algorithm is proposed to identify model parameters. The comparative evaluation demonstrates that the proposed MBW model outperforms the CBW model 66.4% in modeling accuracy, and the self-adaptive cooperative PSO algorithm can obtain the minimum fitness compared with traditional PSO algorithm in parameter identification. At the same time, in order to improve the tracking performance of piezoelectric ceramics, a feedforward control strategy based on MBW model is developed to compensate for the delay and eliminate the error. The position tracking experiment shows that compared with open-loop control and CBW feedforward control, the control strategy proposed in this paper can significantly improve the tracking accuracy of piezoelectric actuators, and effectively achieve accurate control of high-frequency signals. This provides technical guarantee for further improving the precision and machining efficiency of precision machining cutting technology.

## Figures and Tables

**Table 1:** Values of the shape control function for MBW model.

Phase	$\dot{u}$	h	$\ddot{u}$	$\psi (\dot{u},\ddot{u},h)$
1	+	−	+	$\gamma -\beta_{1}+\beta_{2}-\beta_{3}+\beta_{4}-\beta_{5}+\beta_{6}$
2	+	+	+	$\gamma +\beta_{1}+\beta_{2}+\beta_{3}+\beta_{4}+\beta_{5}+\beta_{6}$
3	+	+	−	$\gamma +\beta_{1}-\beta_{2}-\beta_{3}+\beta_{4}+\beta_{5}-\beta_{6}$
4	−	+	−	$\gamma -\beta_{1}+\beta_{2}-\beta_{3}-\beta_{4}+\beta_{5}-\beta_{6}$
5	−	−	−	$\gamma +\beta_{1}+\beta_{2}+\beta_{3}-\beta_{4}-\beta_{5}-\beta_{6}$
6	−	−	+	$\gamma +\beta_{1}-\beta_{2}-\beta_{3}-\beta_{4}-\beta_{5}+\beta_{6}$

**Table 2:** Parameter identification result of CBW model.

Parameters	Value
k	0.106037
A	0.069699
γ	−0.012006
β	0.100000

**Table 3:** Parameter identification result of MBW model.

Parameters	Value
k	0.124131
A	0.062662
γ	0.062490
$\beta_{1}$	0.099500
$\beta_{2}$	−0.003578
$\beta_{3}$	0.083543
$\beta_{4}$	−0.090824
$\beta_{5}$	−0.084294
$\beta_{6}$	−0.050231

**Table 4:** Model performance evaluation.

Model	RMSE (μm)	$err_{\max}\;(\mu m)$
CBW model	0.216506	0.511217
MBW model	0.072782	0.257119

**Table 5:** Parameter identification result with different algorithms.

Algorithms	Traditional PSO (Fixed Inertia Weight)	Traditional PSO (Linear Decreased Inertia Weight)	Modified PSO
Fitness (J)	0.085877	0.080545	0.072782
k	0.120881	0.123564	0.124131
A	0.054375	0.062080	0.062662
γ	−0.011955	0.054668	0.062490
$\beta_{1}$	0.073156	0.067579	0.099500
$\beta_{2}$	−0.022549	−0.00865	−0.003578
$\beta_{3}$	0.014828	0.048177	0.083543
$\beta_{4}$	−0.081405	−0.063953	−0.090824
$\beta_{5}$	0.001384	−0.043963	−0.084294
$\beta_{6}$	0.039550	−0.037160	−0.050231

**Table 6:** Performance of different algorithms in repeat experiments.

Fitness (J)	Traditional PSO (Fixed Inertia Weight)	Traditional PSO (Linear Decreased Inertia Weight)	Modified PSO
Min	0.085877	0.080545	0.072782
Max	0.138687	0.138259	0.133166
Average	0.101663	0.101452	0.090603

**Table 7:** RSME of different control methods under different frequency standard sin signal.

Frequency	Open-Loop Control	CBW Feedforward Compensation	MBW Feedforward Compensation
0.1 Hz	0.674 μm	0.228 μm	0.114 μm
10 Hz	0.693 μm	0.297 μm	0.143 μm
50 Hz	0.952 μm	0.391 μm	0.147 μm
100 Hz	1.112 μm	0.501 μm	0.236 μm

## Data Availability

No new data were created or analyzed in this study. Data sharing is not applicable to this article.
